# Optimal dimensions and performance evaluation of a truncated spherical dielectric lens antenna at X-band frequencies

**DOI:** 10.1371/journal.pone.0318547

**Published:** 2025-03-17

**Authors:** Syed Zeeshan Ali, Kamran Ahsan, Danish ul Khairi, Syed Akhter Raza, Wadee Alhalabi, Lina Hussain Kazem, Muhammad Shahid Anwar

**Affiliations:** 1 Department of Computer Science, Federal Urdu University Arts and Science Technology, Karachi, Sindh, Pakistan; 2 Faculty of Engineering and Computer Science, Millennium Institute of Technology and Entrepreneurship, Karachi, Sindh, Pakistan; 3 Immersive Virtual Reality Research Group, King Abdulaziz University, Jeddah, Saudi Arabia; 4 Department of AI and Software Gachon University Seongnam-si, South Korea; Galgotias College of Engineering and Technology, Greater Noida, INDIA

## Abstract

This study presents the design and development of a truncated spherical dielectric lens antenna. The primary goal of this research is to significantly improve and optimize the directivity and beamwidth characteristics of the feed antenna by adjusting the feed position around the focal point of the lens. Polytetrafluoroethylene (PTFE), with a dielectric constant of 2.1, is chosen as the lens medium. Simulations are carried out using the CST-Studio Suite across the span of 8 to 12 GHz. Vector Network Analyzer (VNA) and an anechoic chamber are utilized for experimental validation. Directivity of 21.9 dBi and a beamwidth of 13.1° have been achieved by optimizing the dimensions of 24.34 mm thickness, 55 mm center of curvature and 110 mm aperture of the lens. By utilizing its simple geometrical shape, compact size, and ease of design and construction, this dielectric lens antenna is ideal for RF applications, particularly when energy concentration is necessary.

## 1 Introduction

High-frequency communication systems employ lenses to send and receive electromagnetic waves (EM). These lenses are evaluated as critical components that provide enhanced efficiency and directivity [1]. In applications such as millimeter (30–300 GHz) and terahertz (0.3–10 THz), dielectric lens antennas are primarily applicable due to their curved surfaces [2]. Initially, the use of dielectric lens antennas, which had been prominent in microwave applications, gradually declined as smaller reflector antennas evolved to become more applicable to microwave configurations [3]. Modern developments in millimeter-wave and submillimeter-wave technologies have reignited interest in antenna design. Due to the essential features of these high-frequency bands, compact and extremely effective antenna solutions are necessary to meet the needs of new applications [4].

Dielectric lenses are frequently used in a variety of applications, such as microwave imaging, broadband communication systems, and radiation pattern optimization employing beam-shaping techniques [5–7]. In contrast with reflectors, dielectric lenses provide a different advantage by enabling beam shaping without blocking the antenna aperture. This characteristic is particularly useful in applications that require high gain while reducing hardware complexity [8]. Integrated lens antennas (ILAs), which feature a direct coupling between the lens base and the feed, are widely used in systems that require high gain and narrow beamwidths. These applications encompass radar, communication, and energy-focusing technologies [9]. Lens antenna subarrays (LAS) are a recent invention in millimeter wave beam guiding technology that significantly reduces hardware complexity [10]. comparebly, horn antenna arrays combined with dielectric lenses, while efficient for multi-beam operation and polarization control, show significant size and enhanced system complexity [11].

state-of-the-art developments have resulted in the fabrication of small, high-performance antennas for X-band and Ku-band applications. A specialized S-shaped slotted micros-trip patch antenna obtains dual-band operation at 10 GHz and 17.65 GHz with a peak gain of 6.3 dB. This antenna’s tiny dimensions (24 × 16 × 0.8 mm³) and enhanced current dispersion are achieved by adding slits and slots. This design demonstrates outstanding performance, utilizing it appropriate for satellite communication and radar systems [12].

The advent of 5G connectivity has drastically increased the need for compact and high-performance antenna designs. A new finding developed a hairpin-shaped micros-trip patch antenna for 5G mobile, operating at 60 GHz and compliant with the IEEE 802.11ad standard. This design was tested on three substrates—FR4, Rogers RO4350B, and Arlon AD255C—This antenna exhibits good bandwidth of 3.8 GHz and a return loss of –34.5 dB when fabricated on a 1.2 mm thick Rogers RO4350B, allowing that it is appropriate for high-frequency applications [13].

To overcome the low directivity of microstrip patch antennas, a novel approach was developed that uses inverse refraction metamaterials (IRMM) as a flat lens. This work proposes an omega-shaped metamaterial (OSM) lens layer specifically designed for Ku-band operation at 12 GHz. The experimental results showed a significant increase in antenna directivity, with a single layer of the OSM lens achieving a 2.74 dB improvement and a double-layer configuration achieving a 4.08 dB improvement. Notably, the metamaterial layers employed in this study have dimensions equivalent to the patch size, indicating that it can be used for compact implementation while getting a considerable performance benefit [14].

Metasurfaces are a promising technology for regulating electromagnetic waves, with features like beam focusing, polarization control, and multi-beam creation [15–17]. Despite these advances, many contemporary designs are hampered by difficulties like as structural complexity, high fabrication costs, and poor adaptation to small hardware systems. Metamaterial-based lenses [18–22] and metasurfaces [23–26] perform well in gain enhancement and beamforming, but require complex geometries and parameter tuning.

For directivity optimization, a truncated PTFE-based spherical lens is significantly more effective than materials including glass or acrylic. PTFE’s low dielectric value and minimum signal loss enhance the high-frequency utilization, while glass’s larger dielectric constant leads to significant signal degradation [27]. Moreover, PTFE is more durable than glass and acrylic because to its greater thermal stability and resistance to environmental conditions such as moisture and UV radiation [28]. Lightweight and flexible, it is perfect for small antenna systems [29]. PTFE lenses are more efficient and adaptable than acrylic, making them promising choice for use in high-frequency antenna systems [30]. With these attributes, PTFE lenses are ideal options for applications that demands precision beam shaping and adaptive radiation patterns.

Part I includes a brief overview of the research aims and essential background information. Part II looks into the lens design process, using the CST Studio Suite software and according to recognized lens optics principles. Key characteristics such as lens thickness (T), radius of curvature (R), lens aperture (L), and focal length (f) were computed methodically. Iterative simulations were run, with parameter tweaks made to attain optimal performance. Part III describes the experimental setup and methodology used for practical demonstrations. Finally, Part IV provides a full analysis of the experimental data, verifying the design and evaluating its performance characteristics.

## 2 The design, optimization, and fabrication of feed and lens antennas

**Fig 1 pone.0318547.g001:**
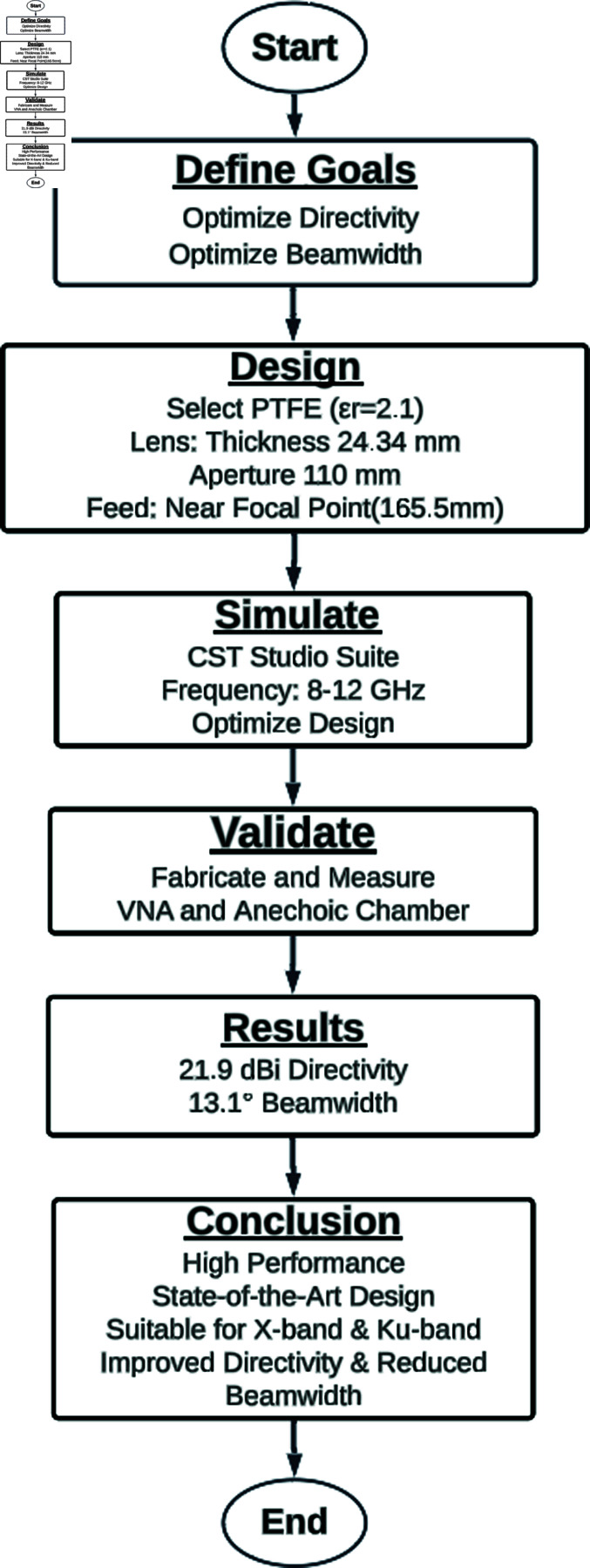
Flowchart of the experimental procedure.

In this section, feed and lens antennas are designed, optimized, and fabricated. Design is guided by feed selection, electromagnetic simulations, and optimizations, followed by fabrication methods that turn the optimized design into a physical structure. Fig 1 illustrates the experimental process.

### 2.1 Designing feed antenna

**Fig 2 pone.0318547.g002:**
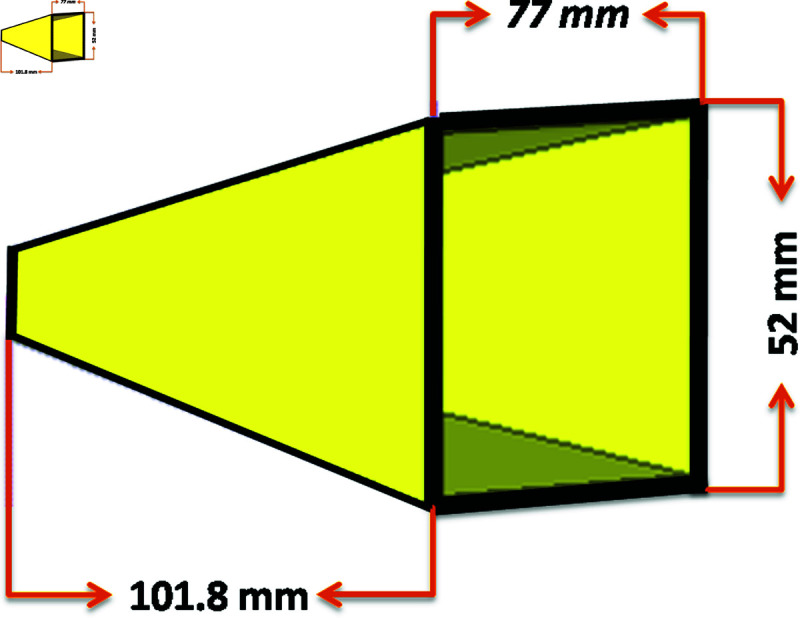
Design view of horn antenna.

To feed the lens, a pyramidal horn antenna with an operating frequency of 11 GHz was chosen. The design parameters of the horn antenna are shown in Fig 2.

**Fig 3 pone.0318547.g003:**
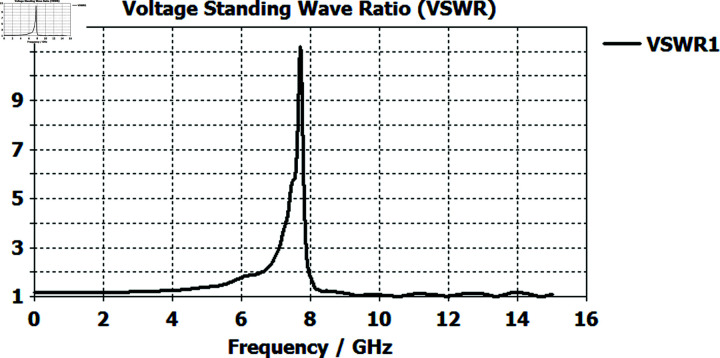
Simulated reflection coefficient graph of the horn antenna, depicting impedance characteristics across the frequency range from 0 GHz to 15 GHz.

The voltage standing wave ratio of the horn antenna is not good for the entire band of 0 GHz to 15 GHz, However, acceptable performance (VSWR < 2) is observed from 8 GHz onwards, as depicted in Fig 3. The simulation range will be refined to 8–12 GHz to ensure optimal feed antenna performance.

**Fig 4 pone.0318547.g004:**
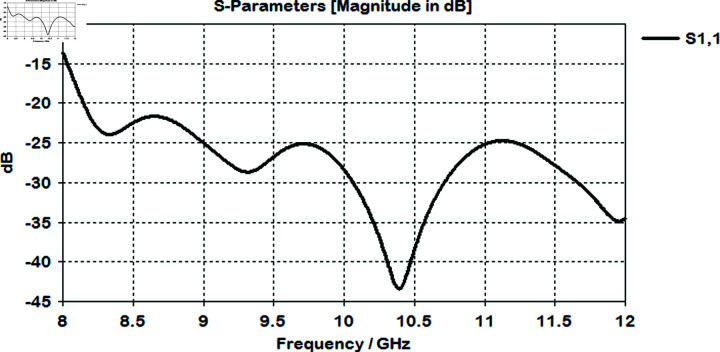
Simulated reflection coefficient graph of the horn antenna, depicting impedance characteristics across the frequency range from 8 GHz to 12 GHz.

The horn antenna was simulated within the CST Studio Suite over the frequency range of 8 GHz to 12 GHz. As depicted in Fig 4, the designed horn antenna exhibits favorable performance with S11 below –10 dB, indicating good impedance matching.

### 2.2 Design and calculation of semi-circular dielectric lens

Taking into consideration of lens shape, it would be based on the refractive index. The refractive index of dielectric materials can be calculated as in Eq 1 [26].


$$\begin{array}{c} n=\sqrt{\varepsilon_{r}} \end{array}$$
(1)


From an optical formula, the relation between focal length and the radius of curvature R of the two lens surfaces can be written as in Eq 2.


$$\begin{array}{c} 1∕f=(n-1)(1∕R_{1}-1∕R_{2}) \end{array}$$
(2)


where a concave surface is indicated by a negative radius. The radius of curvature R of the surface of a single lens is determined using an optical formula, as shown in Eq 3.


$$\begin{array}{c} f=R∕(n-1) \end{array}$$
(3)


Material made of PTFE has a dielectric constant of 2.1 Therefore, Eq 3 may be summed up in Eq 4


$$\begin{array}{c} f=R(0.45) \end{array}$$
(4)


**Fig 5 pone.0318547.g005:**
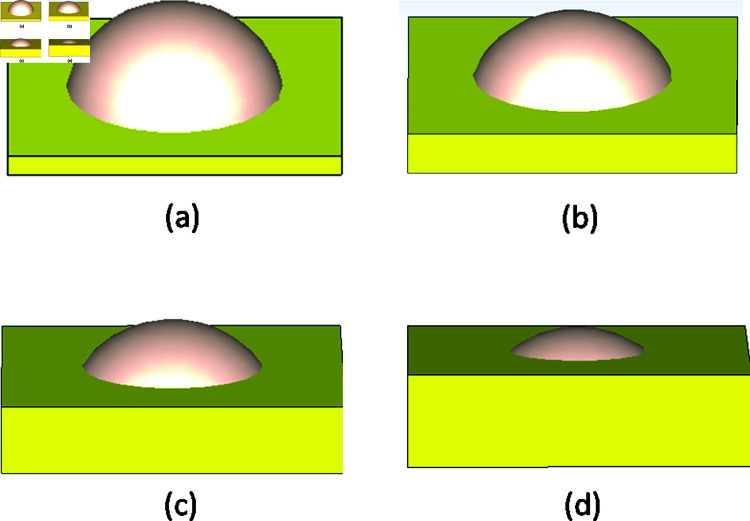
Cutting views of the R = 100 mm radius of the sphere by different sizes of the plan (yellow in color) to get the different thickness of Lens (a) T = 70 mm (b) T = 50 mm (c) T = 25 mm (d) T = 15 mm.

We first fixed the radius of curvature of the lens sphere to R = 100 mm and cut the sphere with a different-sized plane to capture and obtain lenses of different thicknesses and apertures. Fig 5 depicts the entire procedure.

**Fig 6 pone.0318547.g006:**
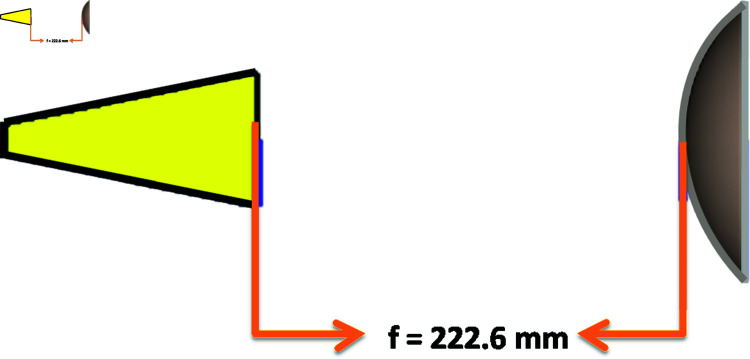
Horn antenna as feed antenna.

By using Eq 4, the Focal length, f = 222.6 mm was calculated at R = 100 mm, and then the feed antenna was placed 222.6 mm away from the lens as shown in Fig 6.

**Fig 7 pone.0318547.g007:**
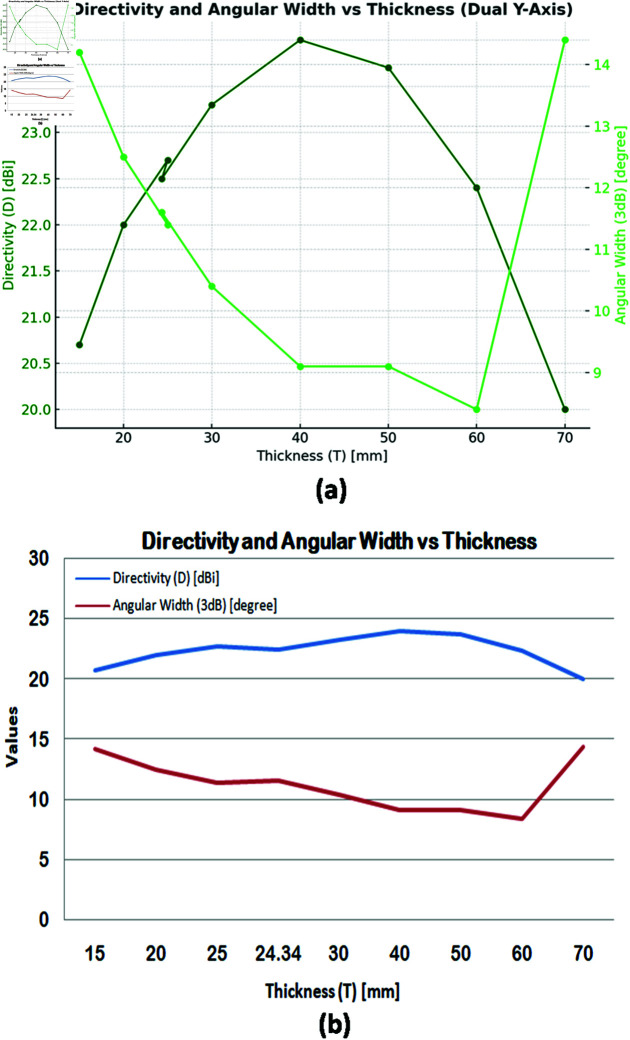
Variation of directivity and angular width with feed position: (a) Graph with dual Y-axes, (b) Graph with single Y-axis.

A simulation-based approach was used to investigate the influence of lens thickness on directivity and angular beam width. Fig 7(a) and 7(b) graphically present the simulation results, offering valuable insights into design optimization. Compared to tabular data, these visual representations effectively convey complex relationships, facilitating the identification of trends and patterns. This approach improves both the efficiency of data analysis and the presentation of research outcomes.

A dual-axis plot in Fig 7(a) shows that as thickness increases, directivity increases steadily, reaching a maximum of 24 dBi at 40 mm. Directivity is observed to diminish with increasing thickness, with a marginal increase observed between T = 25 mm and T = 40 mm (22.7 dBi). With increasing thickness, the angular beam width also narrows, reaching a minimum of 8.4 degrees at T = 60 mm. It is clear, however, that the beam width is 9.1 degrees, demonstrating a reasonable compromise between beam sharpness and thickness at T = 40 mm.these observations indicate that although greater thickness typically enhances directivity and beam focus, the performance gains diminish significantly beyond T = 25 mm, accompanied by increased design complexity and aperture size.

Fig 7(b) confirms these trends, with directivity peaking at T = 40 mm and minimal gains beyond T = 25 mm. Beam width narrows to a minimum at T = 60 mm, remaining adequate at T = 40 mm. T = 25 mm optimizes directivity (22.7 dBi) and beam width (11.4 degrees) while requiring a 132 mm aperture compared to 160 mm at T = 40 mm. The presented data identifies T = 25 mm as the optimal lens thickness, maximizing directivity and beam focus while minimizing aperture size and design complexity. This selection provides a more efficient and cost-effective solution for applications requiring high performance.

Finally, further optimization of the lens was performed with different feed position values around the calculated actual focal length (f = 165.5 mm) as shown in Table 1.

In comparison to alternative feed sites, the data shown in Table 1 shows that placing the feed at f = 165.5 mm results in better directivity and beam width. By increasing the system’s capacity to concentrate radiated energy and reducing the beam width, this result implies that f = 165.5 mm is the best feed site. By positioning the feed in this manner, the system may increase the focus and direction of the energy it radiates, producing a smaller and more focused beam.

**Fig 8 pone.0318547.g008:**
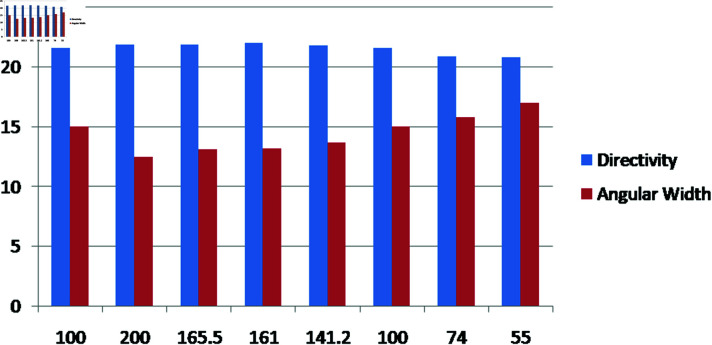
Variation of directivity and angular beam width with lens thickness.

The relationship between the position of the feed, the directivity, and the angular width of a lens is also shown in Fig 8. The bars show the directivity and angular width values for various feed positions, demonstrating how these characteristics vary when the lens’ feed position is changed.

**Fig 9 pone.0318547.g009:**
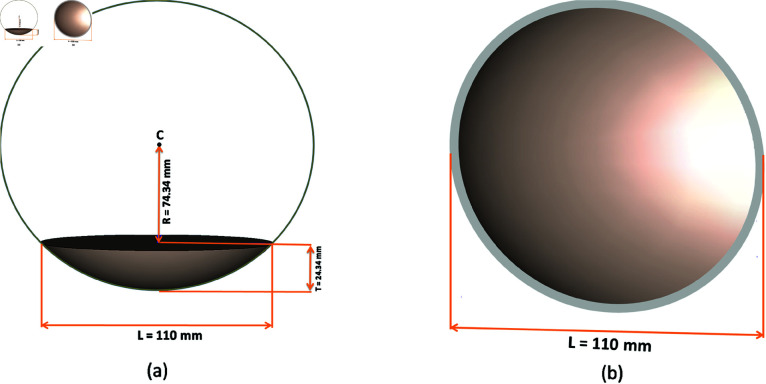
Proposed view of optimized dielectric lens (a) bottom view (b) perspective view.

Hence at f = 165.5 mm, the proposed design geometry and necessary final design values have been shown in Fig 9.

**Table 1 pone.0318547.t001:** Optimized values of directivity and angular width at various positions of feed around the calculated focal length of Lens at R = 74.35 mm and f = 165.5 mm.

S. No.	Thikness of Lens (T) mm	Aperture of Lens (L) mm	Feed position mm	Directivity (D) dBi	Angular Width (3dB) degree
1	25	107.34	100	21.6	15
2	24.34	110	200	21.9	12.5
3	24.34	110	165.5	21.9	13.1
4	24.34	110	161	22	13.2
5	24.34	110	141.2	21.8	13.7
6	24.34	110	100	21.6	15
7	24.34	110	74	20.9	15.8
8	24.34	110	55	20.8	17

In Table 2, which includes a list of the particular lens parameters, you can see the optimized values for each lens parameter that were found through simulation and optimization. Based on the intended result or performance requirements, these optimized values show the optimal or most efficient configuration for the lens.

**Fig 10 pone.0318547.g010:**
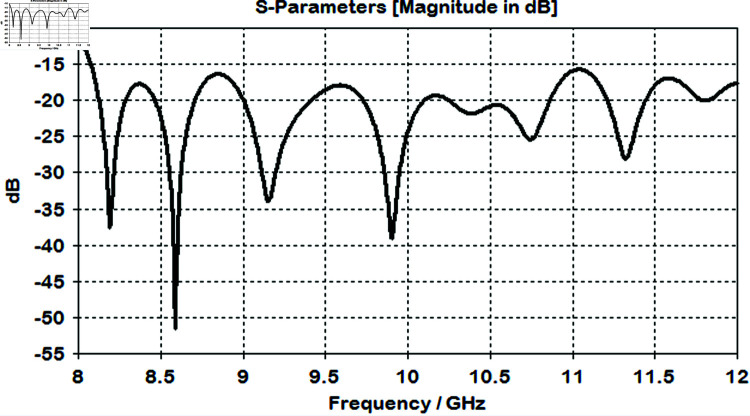
Simulated reflection coefficient graph of horn antenna after placing it infront of lens.

The lens is simulated with optimized values to check the return loss and impedance matching. it is showing good performance with S11  <  –10 dB which indicates that the optimized lens is effectively reducing reflections and improving impedance matching as shown in Fig 10.

**Fig 11 pone.0318547.g011:**
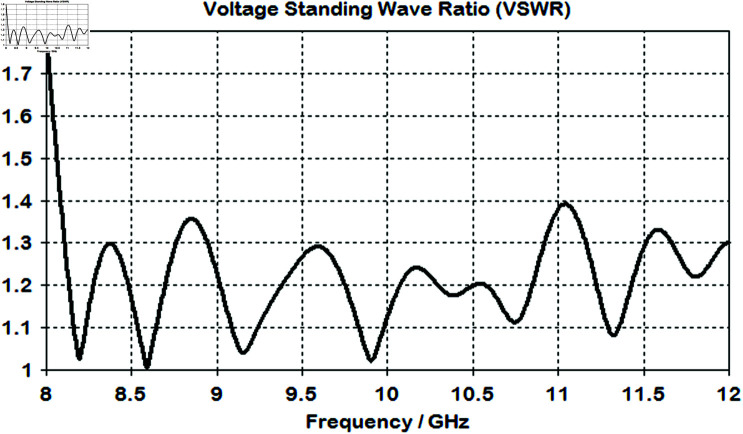
Simulated voltage standing wave ratio (VSWR) of the horn antenna after placing it infront of the lens.

The value of the voltage standing wave ratio of the lens is below 2 for the entire band of interest, as shown in Fig 11.

**Table 2 pone.0318547.t002:** Final optimized values of lens.

S. No.	Lens Parameter	Symbol	Optimized Values (mm)
1	Thickness of Lens	T	24.34
2	Radius of Curvature	R	74.34
3	Aperture of Lens	L	110
4	Focal length	f	165.5

**Fig 12 pone.0318547.g012:**
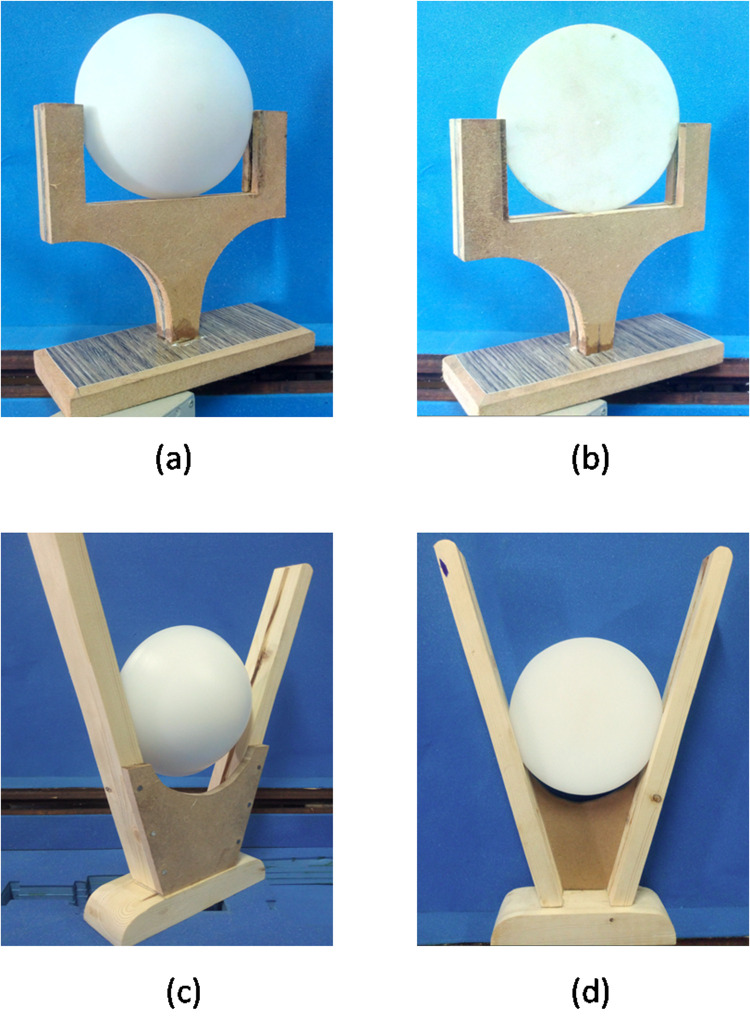
Fabricated lens antenna in wooden frame (a) Front side in a fixed frame (b) Back side in a fixed frame (c) Front Side in a customized frame (d) Back side in customized frame.

The CNC machine was used to manufacture the pair of lenses. In order to physically align the lens center and feed center on a straight line, lens support frames (custom and non-custom) were made to hold the antenna. Further, the lens mount provides a convenient way to move the lens to a different position from the feed point. The lenses in the frames are shown in Fig 12.

## 3 Results and discussion

**Fig 13 pone.0318547.g013:**
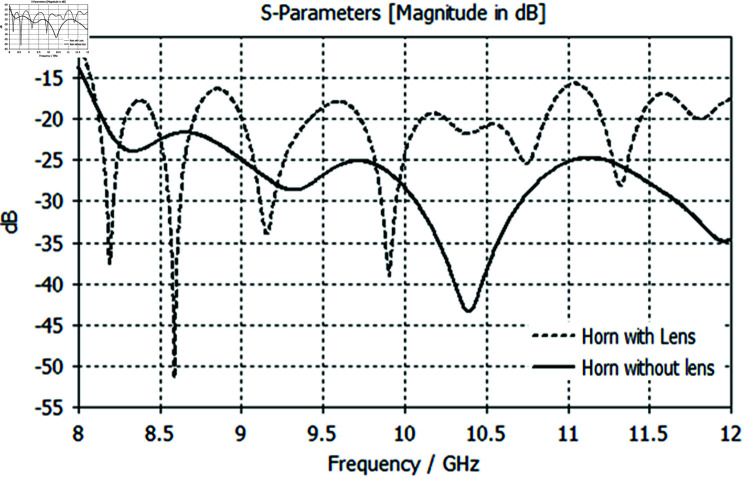
Integrated reflection coefficient graph of the feed and lens showing impedance matching performance from 8 GHz to 12 GHz.

A return loss graph for both the feed and the lens is shown simultaneously in Fig 13. Below S11  <  –10 dB, the graphs of the simulated return loss from 8 to 12 GHz show good performance. A resonance frequency below S11  <  –10 dB is shown by the S11 findings without a lens; however, many resonance frequencies below S11  <  –10 dB are shown by the S11 results with a lens.

**Fig 14 pone.0318547.g014:**
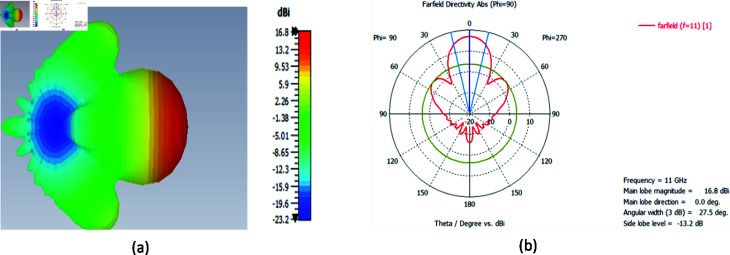
Directivity pattern of the horn antenna at 11 GHz, illustrating the radiation intensity and directional focus (a) 3D plot (b) polar plot.

**Fig 15 pone.0318547.g015:**
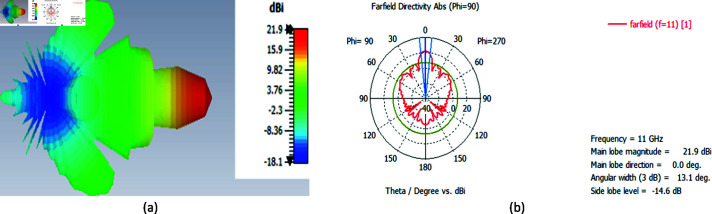
Enhanced directivity pattern achieved by deploying a dielectric lens in the antenna system at 11 GHz (a) 3D Plot (b) Polar Plot.

**Fig 16 pone.0318547.g016:**
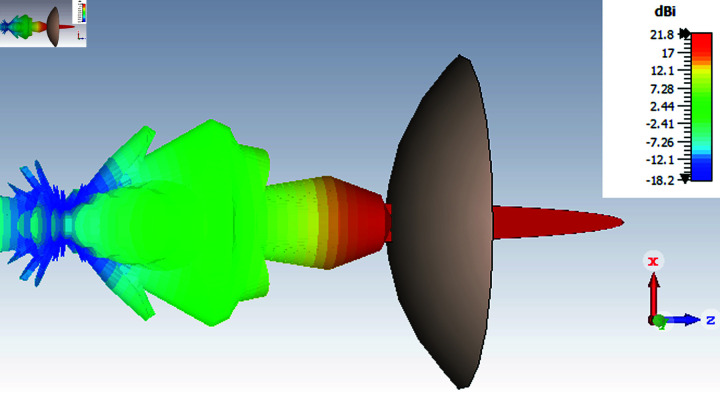
The convergence of energy after deploying dielectric lens.

The far-field results show that the directivity of the horn antenna at 11 GHz is 16.8 dBi and the angular width of the beam is 27.50.^ ∘ ^ as shown in Fig 14(a) and 14(b).

we set our horn antenna to the focal distance of the lens antenna and chose a band between 10GHz to 12GHz. the findings indicate that the horn antenna’s directivity at 11 GHz has been enhanced to 21.9 dBi and the beam’s angular width has been decreased by 13.1^ ∘ ^. As shown in Fig 15(a) and 15(b),

The simulated results, which are depicted in Fig 16, illustrate that the energy concentrates in a certain direction that corresponds to the focal point or peak. At this focal point, when energy intensity is at its peak, the lens has successfully focused and converged energy. In addition, the graph shows the existence of side lobes, which are smaller peaks or secondary maxima seen at angles distant from the focal point. The lens has concentrated some energy in the side lobes, although it was less intense than it was in the focal center. Side lobes may be the consequence of diffraction or flaws in the lens’s construction.

**Fig 17 pone.0318547.g017:**
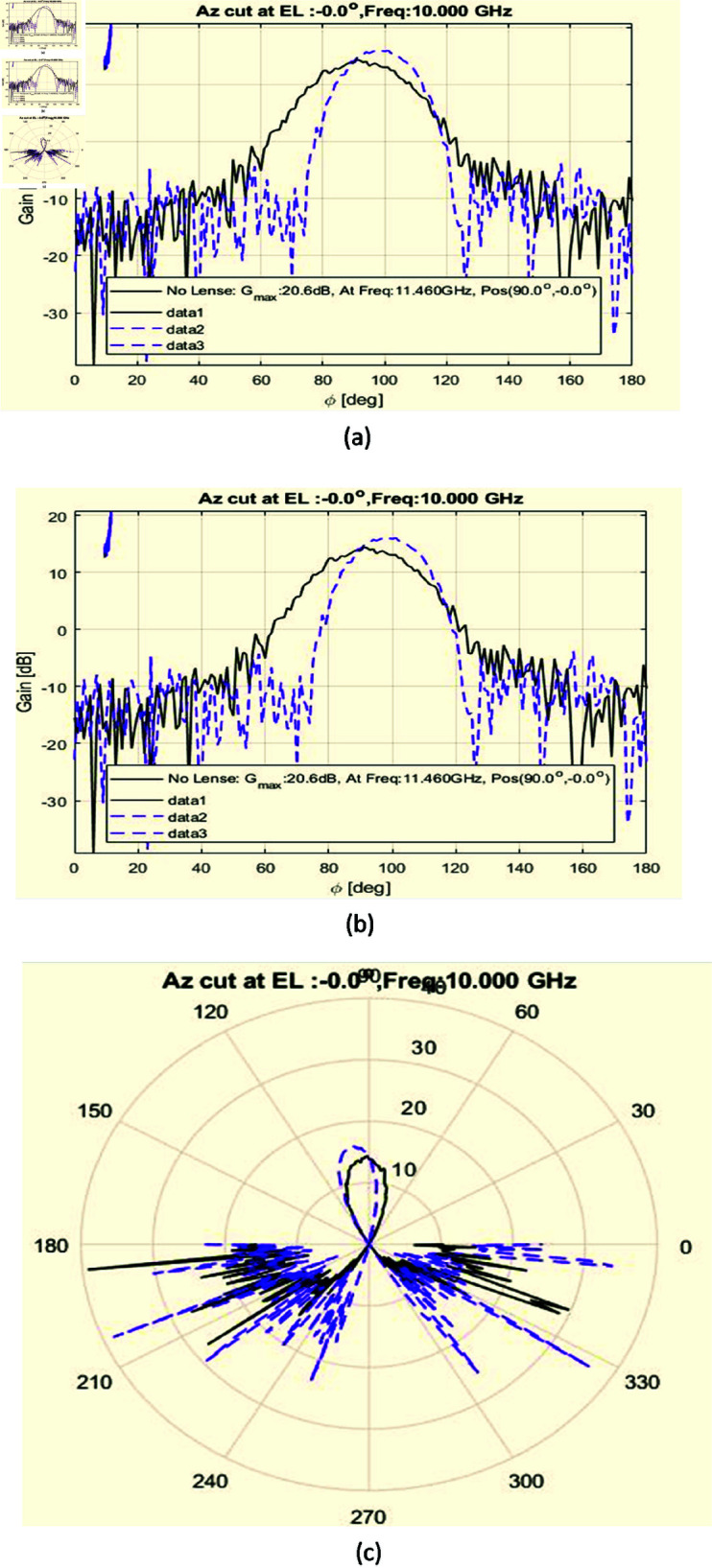
The Measured Integrated results of Directivity (a) Far-field radiation pattern of the antenna with lens, illustrating focused energy and high directivity (b) Radiation pattern of the antenna after passing through the lens, showing energy convergence and focusing towards the focal point (c) Radiation pattern of the antenna with lens, demonstrating focused energy and directional beam.

An anechoic chamber has been utilized to verify the directivity measurement result. The proposed hardware has been placed in the chamber and used a range of 10 GHz to 12 GHz signal by a vector network analyzer. A comparison of the measured response change in directivity with and without the lens has been shown in Fig 17(a), 17(b) and 17(c).

According to 17(a), 17(b), and 17(c), there is a high-intensity focal point or peak as a result of the lens’s effective convergence of the horn antenna energy in a particular direction. As the angular location travels away from the focal point, the energy constricts in this direction as the intensity weakens. The graphs also appear to reveal a primary lobe that is rather narrow and exhibits a marked decrease in energy intensity when the angle is moved away from the focal point. This indicates that the lens has successfully focused and concentrated the energy, resulting in narrow beam width and great directivity.

**Fig18 pone.0318547.g018:**
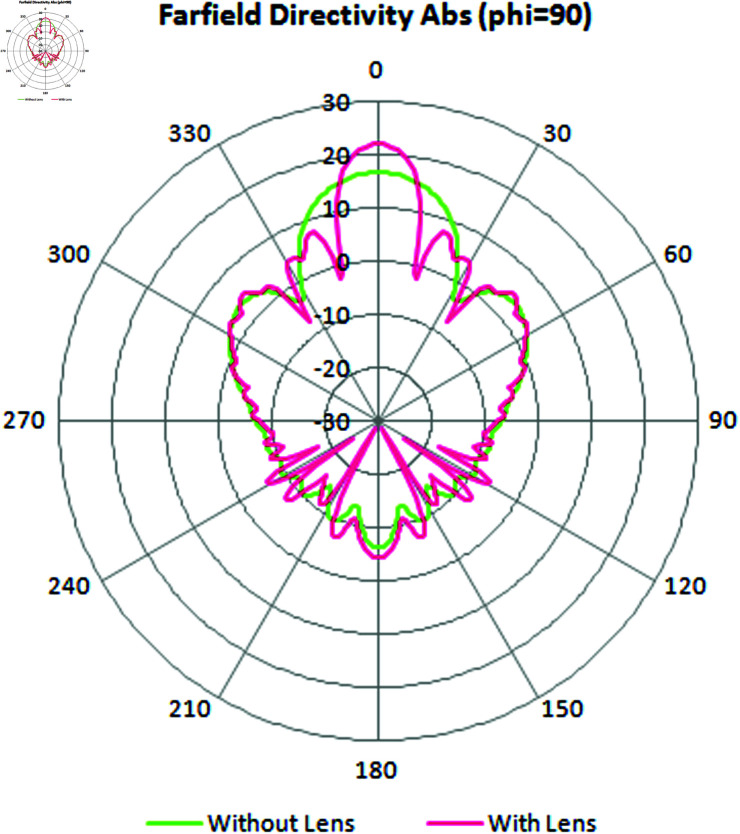
The integrated result of far-field directivity (phi = 90^ ∘ ^).

In Fig 18, the integrated results of the far-field directivity (phi = 90^ ∘ ^) have been shown, and the significant change in directivity and beam-width with and without lens can be observed. it appears that the inclusion of a lens has caused a significant change in the directivity and beamwidth of the antenna in the (phi = 90^ ∘ ^) plane.

## 4 Conclusion

It is not uncommon for the inclusion of a lens to significantly alter the directivity and beamwidth of an antenna, as lenses can be used to focus or defocus the radiated energy in different directions. The specific impact on the directivity and beamwidth will depend on the design and properties of the lens, the antenna, and the operating frequency of the system. The present works have succeeded in designing the dielectric lens antenna

The results of this study demonstrate the usefulness of the truncated directed lens antenna for X-band and Ku-band applications. The design strategy and analytical methods used in this study enhance antenna technology in these frequency bands. The antenna displayed better directivity, allowing it to concentrate and focus the energy radiated in a particular direction, hence boosting signal intensity and coverage in the intended locations. The smaller beamwidth also assured improved spatial resolution and reduced interference from nearby sources. As summarized in Table 3, this lens design achieves superior performance with a simplified structure, wide bandwidth, and enhanced gain, suitable for compact and efficient communication systems.

**Table 3 pone.0318547.t003:** Comparison of dielectric lens designs.

Ref.	Year	Design Complexity	Frequency Band	Directivity (dBi)	Beamwidth (degree)	Gain (dBi)
[31]	2024	Moderate	Wideband	22	12.5	21.7
[32]	2023	High	X-band	21.5	14	20.8
[33]	2023	Moderate	X-band	20.8	14.2	20.4
[34]	2022	Low	X-band	19.8	15.5	19.2
[12]	2023	Moderate	X-band (10 GHz) and Ku-band (17.65 GHz)	–	–	6.3 dB
[13]	2020	Moderate	60 GHz	8.35 (on Arlon AD255C)	–	7.8
[14]	2019	Moderate	12 GHz	7.53 (MPA+OSM)	–	–
**PW**	2024	**Low**	X-band	21.9	13.1	21.5

*PW = Present Work.

## Acknowledgments

I would like to express my special thanks of gratitude to my supervisor Dr Kamran Ahsan as well as all authors who gave me the golden opportunity to do this wonderful project on the topic “Optimal Dimensions and Performance Evaluation of a Truncated Spherical Dielectric Lens Antenna at X-Band Frequencies,” which also helped me in doing a lot of Research and I came to know about so many new things. I am really thankful to them. Secondly, I would also like to thank my parents and friends who helped me a lot in finalizing this project within the limited time frame.

## References
